# Specific fungi associated with response to capsulized fecal microbiota transplantation in patients with active ulcerative colitis

**DOI:** 10.3389/fcimb.2022.1086885

**Published:** 2023-01-05

**Authors:** Qiongyun Chen, Yanyun Fan, Bangzhou Zhang, Changsheng Yan, Zhangran Chen, Lin Wang, Yiqun Hu, Qingwen Huang, Jingling Su, Jianlin Ren, Hongzhi Xu

**Affiliations:** ^1^ Department of Gastroenterology, Zhongshan Hospital of Xiamen University, School of Medicine, Xiamen University, Xiamen, China; ^2^ Institute for Microbial Ecology, School of Medicine, Xiamen University, Xiamen, China; ^3^ Xiamen Key Laboratory of Intestinal Microbiome and Human Health, Zhongshan Hospital of Xiamen University, Xiamen, China; ^4^ Department of Digestive Disease, School of Medicine, Xiamen University, Xiamen, China

**Keywords:** fecal microbiota transplantation, capsule administration, ulcerative colitis, mycobiota, metagenomics

## Abstract

**Objective:**

Fecal microbiota transplantation (FMT) is a novel microbial treatment for patients with ulcerative colitis (UC). In this study, we performed a clinical trial of capsulized FMT in UC patients to determine the association between the gut fungal community and capsulized FMT outcomes.

**Design:**

This study recruited patients with active UC (N = 22) and healthy individuals (donor, N = 9) according to the criteria. The patients received capsulized FMT three times a week. Patient stool samples were collected before (week 0) and after FMT follow-up visits at weeks 1, 4, and 12. Fungal communities were analysed using shotgun metagenomic sequencing.

**Results:**

According to metagenomic analysis, fungal community evenness index was greater in samples collected from patients, and the overall fungal community was clustered among the samples collected from donors. The dominant fungi in fecal samples collected from donors and patients were *Ascomycota* and *Basidiomycota*. However, capsulized FMT ameliorated microbial fungal diversity and altered fungal composition, based on metagenomic analysis of fecal samples collected before and during follow-up visits after capsulized FMT. Fungal diversity decreased in samples collected from patients who achieved remission after capsulized FMT, similar to samples collected from donors. Patients achieving remission after capsulized FMT had specific enrichment of *Kazachstania naganishii*, *Pyricularia grisea*, *Lachancea thermotolerans*, and *Schizosaccharomyces pombe* compared with patients who did not achieve remission. In addition, the relative abundance of *P. grisea* was higher in remission fecal samples during the follow-up visit. Meanwhile, decreased levels of pathobionts, such as *Candida* and *Debaryomyces hansenii*, were associated with remission in patients receiving capsulized FMT.

**Conclusion:**

In the metagenomic analysis of fecal samples from donors and patients with UC receiving capsulized FMT, shifts in gut fungal diversity and composition were associated with capsulized FMT and validated in patients with active UC. We also identified the specific fungi associated with the induction of remission. ClinicalTrails.gov (NCT03426683).

## Introduction

Ulcerative colitis (UC) is a prototypical autoimmune disease with chronic intestinal inflammation (Loftus, 2004; Kaplan and Windsor, 2021). The hallmarks of functional dysregulation in UC are an abnormal mucosal barrier, immune dysregulation, and gut microbiota dysbiosis, which result in inflammation-mediated intestinal destruction (Guan, 2019). Currently, therapeutic regimens for UC aim to regulate immune system disorders and intestinal inflammation, including 5-aminosalicylic acid (5-ASA), sulfasalazine (SASP), corticosteroids, immunosuppressants, and biological agents (Ordas et al., 2012; Khanna et al., 2013). However, these therapeutic regimens are not universally effective and can produce adverse effects. Hormone dependence, long-term maintenance, and relapse are the most common problems that can influence the response to therapy (Ordas et al., 2012). Therefore, it is important to explore practical and safe novel clinical therapies for patients with UC.

Dysbiosis of gut microbiota, including fungi, is associated with many diseases (Ni et al., 2017; Liu et al., 2020; Fan et al., 2021; Liu et al., 2021). Gut fungal communities are often ignored in clinical practice studies because of their low abundance in the intestinal tract (approximately 0.1% of the total microorganisms); however, they are critical components of the human gut microbiota that are essential for human health (Arumugam et al., 2011). Growing evidence has revealed that dysbiosis of intestinal fungi is involved in UC. The biodiversity and composition of fungi are different between healthy individuals and patients with UC, as well as those in remission (Nishikawa et al., 2009; Sokol et al., 2017). These changes also exist between inflamed and non-inflamed intestinal mucosa (Li et al., 2014). Fungi-specific 18S rRNA sequencing analysis of colonic biopsies and fecal samples between UC patients and healthy individuals showed that the fungal community diversity in patients tended to increase, and *Candida albicans* more heavily colonized the patient’s intestinal tract (Ott et al., 2008), causing worse mucosal injury and higher production of anti-*Saccharomyces cerevisiae* antibodies (McKenzie et al., 1990; Colombel et al., 2013). An increased abundance of pathogenic fungi, such as *Trichosporon*, and a lower abundance of *Saccharomyces* were found in the colonic mucosa in severe dextran sodium sulfate (DSS)-induced colitis in mice (Qiu et al., 2015), also demonstrating a close relationship between fungal dysbiosis and UC. Another studies showed that Dectin-1-deficient mice were more susceptible to DSS-induced colitis because of the aberrant immune response to the host fungus (Goodridge et al., 2011; Iliev et al., 2012). Furthermore, UC patients with severe intestinal damage are associated with a polymorphism in the gene encoding Dectin-1 (CLEC7A) (Iliev et al., 2012). Although fungal dysbiosis has been found in patients with UC and some fungi can regulate the intestinal inflammatory response (Calich et al., 2008; Ott et al., 2008; Sonoyama et al., 2011; Sokol et al., 2017; Wang et al., 2019), it is unclear whether the change in fungal composition is a result or cause of UC. Moreover, the functional roles of fungi remain largely unknown.

Fecal microbiota transplantation (FMT) is a strategy to reconstruct gut microbiota by administering a new microbiome from healthy individuals to patients (Ooijevaar et al., 2019). It has been proven that FMT partly works in patients with UC (Moayyedi et al., 2015; Costello et al., 2019; Crothers et al., 2021; Haifer et al., 2022). In contrast to the great success of FMT in treating recurrent *Clostridium difficile* infection (CDI), heterogeneous responses to FMT have been reported in patients with UC (Borody et al., 2003; Smits et al., 2013; Paramsothy et al., 2017). A systemic study including 122 patients with IBD found that approximately 22% of patients with UC achieved clinical remission after FMT during a follow-up visit (Colman and Rubin, 2014). However, compared with numerous studies on the influence of FMT on bacteria (Rossen et al., 2015; Li et al., 2016; Mintz et al., 2018; Paramsothy et al., 2019; Huang et al., 2022), little is known about the alterations in fungal communities after FMT. Siew et al. recently reported durable engraftment of donor-derived fungi after FMT in graft-versus-host disease (Zhang et al., 2021). Another study also revealed that fungal species were associated with the efficacy of FMT in recurrent CDI (Zuo et al., 2018). In 2020, Irina et al. found that *Candida* colonization may be associated with the clinical outcomes of FMT in UC patients (Leonardi et al., 2020). In addition, experimental studies have revealed the effects of gut *Candida* on intestinal inflammation (Jawhara et al., 2008; Leonardi et al., 2018; Sovran et al., 2018; Li et al., 2019). These changes imply that alterations in gut fungi after FMT may be important for clinical outcomes. However, more evidence is needed to elucidate the function of fungi associated with the response to FMT in UC.

In this study, we used shotgun metagenomics to analyze the longitudinal dynamic change of gut fungal communities in active UC patients after capsulized FMT treatment. We demonstrated significant alterations in the fungal community in patients who received capsulized FMT and identified specific fungi associated with clinical remission after FMT.

## Material and methods

### Study design

We recruited volunteers as healthy donors from Xiamen University. After screening questionnaires and medical examinations to exclude disease risks, we finally obtained nine volunteers to provide healthy fecal microbiota. After applying the inclusion and exclusion criteria, 22 patients with active UC were enrolled. Before capsulized FMT, all patients underwent endoscopies to assess the severity of GI mucosal to calculate the Mayo scores. Capsulized FMT was administered three times and followed up for 12 weeks. We collected stool samples at weeks 0, 1, 4, and 12 for fungal analysis. **Figure S1** showed the flow diagram of the study. This study was approved by Zhongshan Hospital of Xiamen University and registered in ClinicalTrials.gov (NCT03426683).

### Donor management

Donors were recruited by advertisement from Xiamen University. Screening questionnaires, including lifestyle and medical history interviews, were undertaken before blood and fecal tests. According to the European consensus conference on FMT (Cammarota et al., 2017), the donor’s medical history screened for the following criteria with subsequent excluded from donating if present: personal history of cigarette smoking, drinking; drug use in the last 1 month including antibiotics, probiotics, probiotics, and any other drugs; personal history of any infection, such as hepatitis, HIV; gastrointestinal diseases, such as functional gastrointestinal disorders, infections, polyps; autoimmune disease, metabolic syndrome, depression, and other system diseases. Blood screening: full blood count, urine routine, liver function tests, urea and creatinine, CRP, ESR, HIV, Hepatitis A, B and C, Epstein-Barr virus (IgG, IgM), tumor markers, TORCH, cytomegalovirus (IgG, IgM), H. pylori (IgG). Stool screening: fecal occult blood test, routine enteric pathogens test, C. difficile toxin B.

### Participant eligibility

Eligible patients were aged 18–70 years with active UC (total Mayo score of 4 - 12) and had a poor response to their current medications (including 5-ASA, SASP, and prednisone). Key exclusion criteria were as follows (Costello et al., 2019; Paramsothy et al., 2019): indeterminate colitis or proctitis alone; other causes of diarrhea; antibiotics or probiotics use within 4 weeks of the enrollment; contraindications for gastrointestinal endoscopy; other diseases such as respiratory failure, heart failure, and severe immunodeficiency; gastrointestinal infection; and other diseases associated with microbiota, such as hypertension, diabetes, systemic lupus erythematosus (SLE).

### FMT capsule preparation

Fresh stool samples from donors were collected in sterile plastic containers. Fresh fecal samples (25%) were mixed with saline (60%) and pharmaceutical-grade glycerol (15%) and filtered using an automatic extraction instrument (TG-01, Treatgut Corporation, China). Microbial suspensions were further centrifuged to obtain the fecal microbiota precipitates. The precipitates were thoroughly mixed and filled into capsules (DrCaps, 19504907), and the capsules were immediately frozen at −80°C. The mean stool per capsule was 0.9 grams.

### Capsulized FMT

All patients provided written informed consent. Authorized physicians administered FMT in a monitored clinical setting. Patients should fast for at least 8 hours prior to FMT. The frozen capsules were removed to room temperature before use. After administration, patients were onwards observed for adverse effects. Enrolled patients received three FMT treatments (once every two days) in a single dose of 30 capsules. The donors were randomly assigned to enrolled patients. Each patient received two donors flora at least. Patients were followed up for 12 weeks after FMT treatment, and their stool samples were collected at weeks 0, 1, 4, and 12. At baseline (W0, before taking FMT) and W12, coloscopies were taken to assess patients’ response to FMT. Maintenance therapy was required in this study according to their usage before enrollment: 1) 5-ASA or SASP- kept using the stable dosage of 5-ASA or SASP; 2) prednisone- with a mandatory taper of 5 mg (>10mg/d) or 2.5mg (≤10mg/d) per week. The weekly time points were abbreviated as W0 (baseline, week 0), W1 (week 1), W4 (week 4), and W12 (week 12).

### Assessment

The efficacy of capsulized FMT was assessed using the Mayo scores (Paramsothy et al., 2017) at W0 and W12. The total Mayo score was 0 to 12, including stool frequency (0 to 3), rectal bleeding (0 to 3), findings on endoscopy (0 to 3), and physician’s global assessment (0 to 3). The primary outcome was patients’ remission (Rm) at W12, with the total Mayo score ≤ 2. Patients could withdraw from the study at any time. To ensure the reliability of data, patients need to contact clinicians before withdrawal and provide their reasons if possible.

### Metagenomic sequencing

Fresh fecal samples were collected and immediately frozen at −80°C. Total fecal DNA was extracted using QIAamp Fast DNA Stool Mini Kit (Qiagen, Germany). After the detection of concentrations of DNA samples by Qubit 3.0 (Thermo Fisher Scientific, USA), the quality was further checked on a 1.5% agarose gel. Next, 100 ng DNA was cut into fragments with an average size of 350 bp using the NEBNext^®^ Ultra™ II DNA Library Prep Kit for Illumina (New England Biolabs, USA). We diluted the library to 1 ng/μL and checked the insert size with Agilent 2100 (Agilent, USA). The effective concentration of the library was accurately quantified using an ABI 7300 Plus (Thermo Fisher Scientific, USA) fluorescent quantitative PCR instrument. Finally, the library was sequenced on an Illumina Novaseq 6000 (Illumina, Inc., San Diego, CA, USA) using PE150 reagents. On the average, 84,594,918 ± 19,431,972 raw paired-end reads per sample were obtained in this study.

### Bioinformatic and statistical analysis

We used Trimmomatic software (Bolger et al., 2014) to delete unqualified reads and sequencing adaptors from raw sequencing reads. The data was also filtered using knead data (https://huttenhower.sph.harvard.edu/kneaddata) to remove the human host’s contaminants. Consequently, 56,668,575 ± 28,151,279 high-quality reads were obtained and subjected to Kraken (Wood et al., 2019) for fungal classification, resulting 21,946 ± 14,082 fungal reads per sample. We utilized the vegan package to analyze fungal α-diversity metrics, Bray-Curtis distances by the vegdist function, and PERMANOVA with 999 permutations by the adonis function (Oksanen et al., 2015). We further used the agricolae package to analyze the diversity indices and taxa among groups by a nonparametric Kruskal-Wallis rank-sum test and Benjamini-Hochberg corrections (Mendiburu, 2015). Figures were plotted using ggplot2-v3.0.0 (Wickham et al., 2017) and GraphPad Prism 9. Statistical significance is represented by *p* value (≤ 0.05). *p ≤ 0.05, **p ≤0.01, and ***p ≤0.001.

## Results

### Gut fungal communities of donors

**Figure 1:**
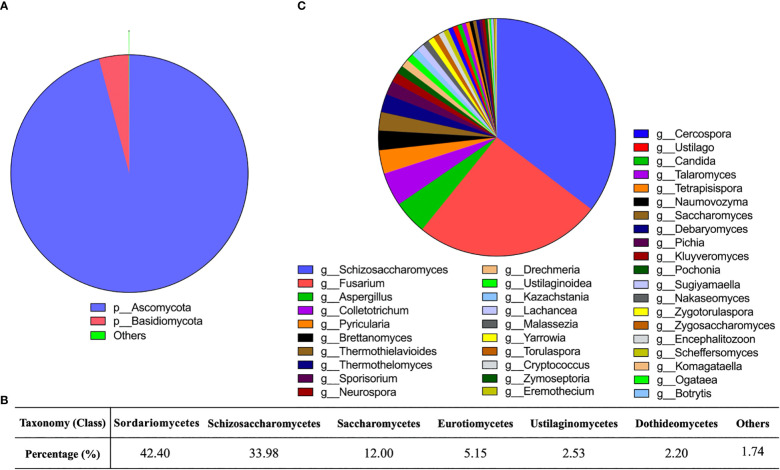
Gut fungal community in the donors. **(A)** Sector diagram showed the overall fungal community structure at the phylum level. **(B)** The percentage of different fungi at the class level. **(C)** Sector diagram showed the composition of the fungal genera.

Gut fungal communities of the donor samples were profiled at different classification levels. We found that at the phylum level, *Ascomycota* and *Basidiomycota* were the predominant fungi in fecal samples, especially *Ascomycota*. The relative abundance of *Ascomycota* was as high as 95% (**Figure 1A**). Ten classes were detected (**Figure 1B**) as follows: *Sordariomycetes* (42.40%), *Schizosaccharomycetes* (33.98%), *Saccharomycetes* (12.00%), *Eurotiomycetes* (5.15%), *Ustilaginomycetes* (2.53%), *Dothideomycetes* (2.20%), *Malasseziomycetes* (0.85%), *Tremellomycetes* (0.72%), and *Leotiomycetes* (0.17%). We had identified 40 fungal genera in donor samples, including *Schizosaccharomyces*, *Fusarium*, *Aspergillus*, *and Colletotrichum*. Among them, the relative abundance of 13 genera was above 1%, and *Schizosaccharomyces* (35.35%) and *Fusarium* (25.52%) were the main genera (**Figure 1C**).

### Gut fungal dysbiosis in patients with UC

**Figure 2:**
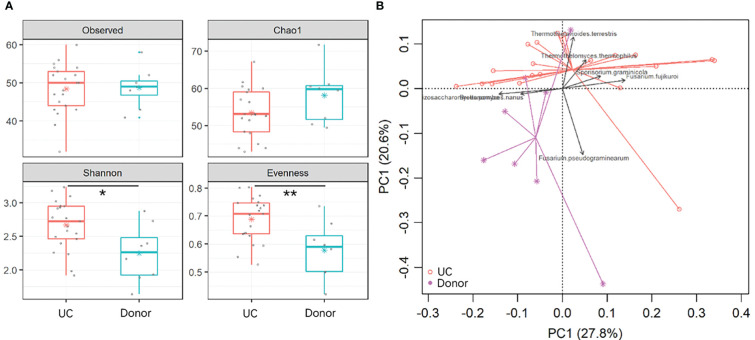
Dysbiosis of gut fungal communities in UC patients. **(A)** α-diversity indices in UC patients and donors were analyzed, including observed OTUs, Chao 1, and Shannon and Pielou’s evenness indices. The asterisk indicated statistical differences between the two groups, *p ≤ 0.05,**p ≤ 0.01,. **(B)** Clusters of gut fungal communities were analyzed using PCA of the fungal species by the Euclidean distance. The top 7 species were fitted to PCA with a significance cutoff at *p* < 0.05.

To reveal gut fungal dysbiosis in patients, α-diversity was analyzed between the donor and UC patients, including observed richness, Chao 1, and Shannon and Pielou’s evenness indices. The results showed that Shannon and Pielou’s evenness indices were significantly increased in samples from UC patients. But Chao 1 richness indice showed a slight decrease in samples from UC patients without a *p*-value (*p* = 0.16) (**Figure 2A**). The p-values for the different indices are shown in **Table 1**. *β*-diversity analyzed using principal component analysis (PCA) revealed that the overall fungal community in patients with UC was significantly clustered from the donor samples (PERMANOVA, F = 2.413, *p* < 0.05). This change was significantly associated with the abundance of *Thermothielavioides terrestris*, *Fusarium fujikuroi*, *Thermothelomyces thermophilus*, *Sporisorium graminicola*, *Brettanomyces nanus*, *F. pseudograminearum*, and *Schizosaccharomyces pombe* (**Figure 2B**).

**Table 1:** The analysis of α-diversity.

Parameters	P value
Chao 1	0.16
Observed	0.86
Shannon	0.02
Evenness	0.01
Simpson	0.02

### Changes in fungi in patients with UC

**Figure 3:**
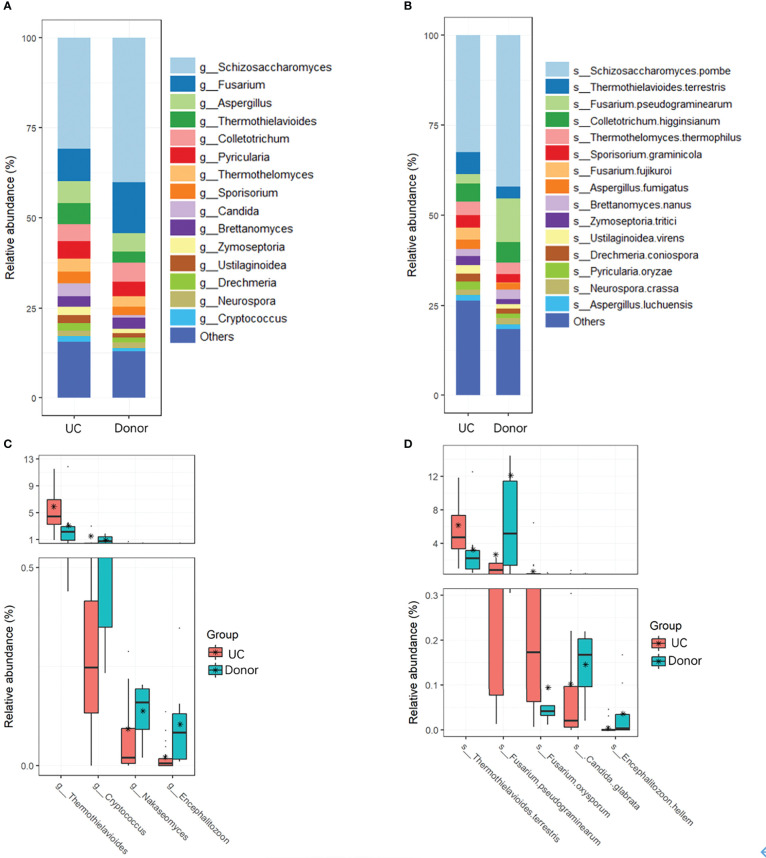
Changes in fungi in UC patients. Overall fungal community structures at the genera **(A)** and species **(B)** levels. **(C)** Relative abundances of genera **(C)** and species **(D)** were significantly different between UC patients and donors.

Fungal taxa at different classification levels were compared between the donor and patient fecal samples to determine the specific changes in gut fungal communities. As expected, *Ascomycota* was the dominant fungal phylotype in patients with UC at the phylum level (**Figure S2**). To further explore differences in the composition of the two groups of fungi, the top 15 dominant genera, namely, *Schizosaccharomyces*, *Fusarium*, *Aspergillus*, *Thermothielavioides*, *Colletotrichum*, *Pyricularia*, *Thermothelomyces*, *Candida*, *Sporisorium*, *Brettanomyces*, *Zymoseptoria*, *Ustilaginoidea*, *Drechmeria*, *Neurospora*, and *Cryptococcus*, were analyzed at the genus level (**Figure 3A**; **Table 2**). However, compared with the donor samples, only *Thermothielavioide* was significantly increased in patients with UC, whereas *Cryptococcus*, *Nakaseomyces*, and *Encephalitozoon* were significantly decreased (**Figure 3C**). The shift in the fungal community of patients was more remarkable at the species level. Consistent with the phylum level, *S. pombe* was the dominant species in both the donor and patient fecal samples (**Figure 3B**). As **Figure 3D** showed five species showed significant changes, including *Thermothielavioide terrestris*, *F. pseudograminearum*, *F. oxysporum, C. glabrata*, and *Encephalitozoon hellem*. Notably, the relative abundance of *F. pseudograminearum* decreased from 23.76% in donor samples to 2.65% in patient samples (**Table 2**). Furthermore, the relative abundance of *T. terrestris* and *F. oxysporum* markedly increased in UC patients, while the relative abundance of *C. glabrata* and *Encephalitozoon hellem* significantly decreased. Unlike the predominant fungi, the change of fungi with low abundance was more remarkable (**Figures 3B, D**). These findings suggested that fungi with low abundance might be of concern in patients with UC.

**Table 2:** Comparison of the main taxonomy of fungi in donor and UC patients.

Taxonomy (Genus)	Donor	UC	P value	Taxonomy (Species)	Donor	UC	P value
g Schizosaccharomyces	35.35	30.75	0.4164	S Schizosaccharomyces pombe	36.86	32.41	0.8193
g Fusarium	25.52	9.12	0.0721	S Thermothielavioides terrestris	2.64	6.16	0.0031
g Aspergillus	4.62	5.99	0.1725	S Colletotrichum higginsianum	4.81	4.99	0.6930
g Thermothielavioides	2.51	5.90	0.0031	S Thermothelomyces thermophilus	2.60	3.77	0.0654
g Colletotrichum	3.22	4.85	0.6930	S Sporisorium graminicola	1.98	3.40	0.1594
g Pyricularia	4.57	4.76	0.2011	S Fusarium fujikuroi	0.16	3.32	0.0194
g Thermothelomyces	0.62	3.69	0.0793	S Fusarium pseudograminearum	23.76	2.65	0.0013
g Candida	2.47	3.60	0.0592	S Aspergillus fumigatus	1.66	2.63	0.1594
g Sporisorium	1.90	3.11	0.1725	S Zymoseptoria tritici	1.14	2.43	0.1470
g Brettanomyces	2.67	2.84	0.9503	S Ustilaginoidea virens	1.05	2.34	0.0311
g Zymoseptoria	1.09	2.33	0.1864	S Pyricularia oryzae	1.02	2.24	0.0101
g Ustilaginoidea	1.01	2.24	0.0348	S Drechmeria coniospora	1.14	2.21	0.0247
g Drechmeria	1.08	2.11	0.0247	S Brettanomyces nanus	2.41	2.00	0.5189
g Neurospora	0.75	1.50	0.1244	S Neurospora crassa	1.43	1.55	0.0955
g Cryptococcus	1.35	1.49	0.2166	S Aspergillus luchuensis	1.00	1.54	0.2866

### Changes of gut fungal communities in patients after FMT

**Figure 4:**
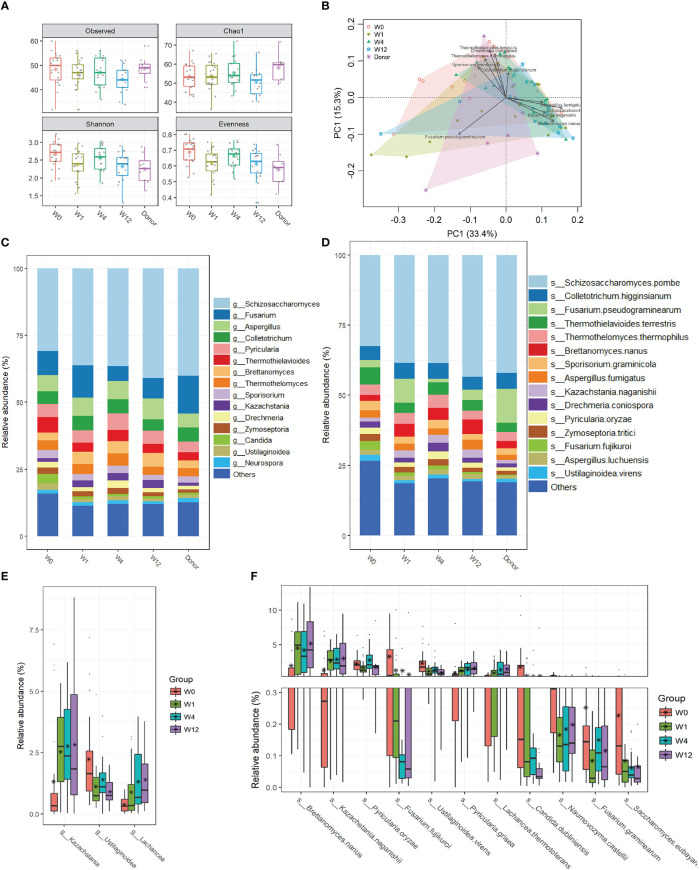
Changes of gut fungal communities of UC patients after FMT. **(A)** α-diversity indices in UC patients were analyzed during the follow-up visits, including observed OTUs, Chao 1, and Shannon and Pielou’s evenness indices. **(B)** Clusters of gut fungal community were analyzed using PCA of the Euclidean distance of OTU abundance. The top ten genera were fitted to PCA with a statistical significance of *p* < 0.05. Relative abundances of the top 15 genus **(C)** and species **(D)** in samples from donors and patients before and after FMT. **(E)** The significant change in relative abundances of genera in samples from patients between week 0 and other time points after FMT. **(F)** The significant change in relative abundances of species in samples from patients between week 0 and other time points after FMT.

To explore the effect of capsulized FMT on gut fungal communities, we profiled the fungal community structures at all time points (W0, W1, W4, and W12) during follow-up visits after capsulized FMT. We analyzed the fungal α-diversity before and after capsulized FMT. There were no obvious changes in the fungal richness of fecal samples after FMT. However, compared with W0, the Shannon and Evenness diversities significantly decreased in samples from UC patients at weeks 1, 4, and 12, which was comparable to that in samples from donor (**Figures 4A**, **S3**). PCA results showed that the overall microbial communities of the patient samples changed after FMT compared to W0 (**Figure 4B**). Notably, the dysbiosis of fungal profiles of patients was restored after FMT, which was more similar to that in donor samples (**Figure 4C**). *Kazachstania and Lachancea* were significantly enriched after FMT, while the relative abundance of *Ustilaginoidea* was decreased at the genus level (**Figure 4E**). In addition, we found more remarkable shifts in microbial profiles at the species level (**Figure 4D**). It was worth noting that the relative abundance of some pathogens (*F. fujikuroi* and *C. dubliniensis*) significantly decreased after FMT (**Figure 4F**). Simultaneously, the relative abundance of *B. nanus*, *Kazachstania naganishii*, *Pyricularia grisea*, and *Lachancea thermotolerans* increased during follow-up visits.

### Decreased pathobiont levels associated with the clinical response to FMT

**Figure 5:**
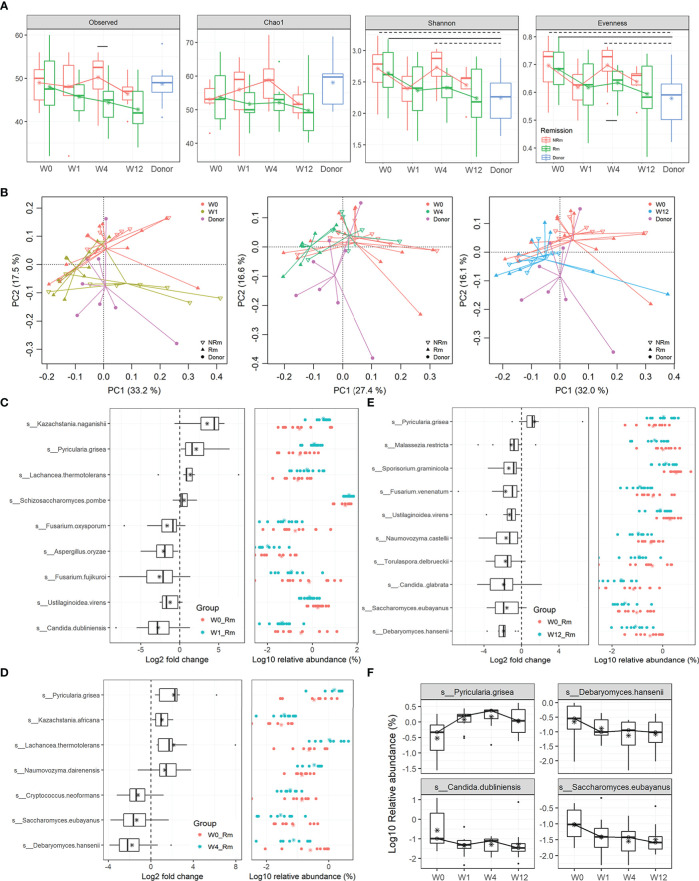
Alterations in gut fungal taxa associated with Rm after FMT. **(A)** α-diversity indices in samples from donors and patients who received remission (Rm) or not (NRm) were analyzed, including Observed species, Chao 1, Shannon, and Pielou’s evenness. **(B)** Clusters of gut fungal community were analyzed using PCA of the Euclidean distance of OTU abundance. The circle represented the donor, the triangle represented patients who received remission (Rm), and the inverted triangle represented patients who did not receive remission (NRm). **(C–E)** The changes in relative abundances of fungal species were assessed by the paired rank-sum test between week 0 and other time points (weeks 1, 4, and 12) (*p* < 0.05). Box plots showed the species significantly changed in the Rm group at different time points. **(F)** The relative abundance of fungal species in remission patients at different time points.

In this study, 22 patients with active UC underwent capsulized FMT, and we assessed the primary outcome at W12 after capsulized FMT. During the follow-up visit, one patient withdrew because of a failed communication. Patients with a total Mayo score ≤ 2 at W12 were defined as clinical Rm (**Figure S4**). Twelve patients received clinical remission at week 12 (12 out of 21). Shotgun sequencing revealed differences in the fungal community in patients who achieved Rm and those who did not (NRm), especially at W4. Compared to W0, the α-diversity analysis showed that the diversity of the fungal community decreased in remission patient samples (Rm) at weeks 1, 4, and 12, similar to that in donor samples (**Figure 5A**). However, compared to W0, Shannon and Pilou’s evenness indices for non-remission patient samples (NRm) decreased at weeks 1 and 12 and increased at week 4. Moreover, Pilou’s evenness index showed a significant difference between Rm and NRm patients at W4 after capsulized FMT (**Figure 5A**).

Fungal communities visualized using PCA exhibited differences between Rm and NRm samples after capsulized FMT (**Figure 5B**). Rm patients clustered tightly since W1, unlike NRm patients. Rm patients who received capsulized FMT also tended to have a steady situation on the left side of the donor cluster and were closer to it. Interestingly, the cluster of NRm patients became tighter and moved closer to the cluster of Rm patients who received capsulized FMT.

To identify specific fungi associated with clinical remission, paired rank sum tests were used to analyze the change in abundance of each species between W0 and other time points. The most distinguished taxa are shown in **Figures 5C–E**. The relative abundance of *K. naganishii*, *P. grisea*, *L. thermotolerans*, and *S. pombe* was increased at W1 after capsulized FMT, whereas *F. oxysporum*, *Aspergillus oryzae*, *F. fujikuroi*, *C. dubliniensis*, and *Ustilaginoidea virens* were decreased (**Figure 5C**). The relative abundance of *P. grisea* remained high abundance at weeks 1, 4, and 12 (**Figures 5C–E**). Interestingly, the relative abundance of *Debaryomyces hansenii*, which could injure intestinal mucosal, was significantly decreased in samples that achieved Rm after capsulized FMT at weeks 4 and 12 (**Figures 5C, D**). We also found that the relative abundance of *Candida dubliniensis* and *Candida glabrata* was decreased at weeks 1 and 12 (**Figures 5C, D**). Furthermore, the relative abundance of most of the specific taxa decreased at W12, except for the increased level in *P. grisea* (**Figure 5E**). The changes of these specific fungi at different time points were shown in **Figure 5F**. These changes might be a sign of rebalancing gut dysbiosis.

## Discussion

In this study, we focused on the gut dysbiosis of fungal communities in patients with UC and conducted capsulized FMT to explore the effect of capsulized FMT on gut fungal diversity and composition. Capsulized FMT reconstructed gut fungal communities in patients with UC. Meanwhile, specific fungal changes from capsulized FMT in UC have been identified and associated with the primary clinical outcome. Improvements in microbial richness, depletion of purported pathobionts (*Candida* and *D. hanseni*), and enrichment of fungal microbes (*K. naganishii*, *P. grisea*, *L. thermotolerans*, and *S. pombe*) were associated with the therapeutic effects of capsulized FMT.

There is growing evidence that gut fungal dysbiosis is associated with UC, even varying in composition in the inflamed or non-inflamed intestinal tract. As reported, the diversity of the fungal community was inconsistent in different types of samples from patients with UC (Ott et al., 2008; Hoffmann et al., 2013; Sokol et al., 2017). Our results showed increased diversity in patient fecal samples based on shotgun sequencing, mainly in microbiota evenness (Shannon and Pilou’s evenness). For the Chao 1 index, fungal biodiversity in UC nominally decreased compared with donor samples, without reaching statistical significance. After capsulized FMT, the fungal microbiota evenness index in patients with UC decreased significantly and was closer to that of donors. Notably, these changes were more remarkable in Rm patients after capsulized FMT than in NRm patients. The borderline differences may be due to limitations related to the sample size of this study.

Following published reports, *Ascomycota* and *Basidiomycota* were the dominant fungal fecal microbiota in both donors and UC patients (Hoffmann et al., 2013; Chehoud et al., 2015; Mukherjee et al., 2015; Richard et al., 2015). We also identified specific fungal community dysbiosis in patients with UC belonging to the dominant phyla *Ascomycota* and *Basidiomycota*. In addition, capsulized FMT reversed the imbalance between *Ascomycota* and *Basidiomycota*. The abundance of *Ascomycota* and *Basidiomycota* was strongly associated with the disease status and also different in biopsies from the inflamed or non-inflamed intestinal tract. Sokol et al. reported that *Ascomycota* and *Basidiomycota*, especially the Basidiomycota-to-Ascomycota ratio, were two of the most distinguishing features between healthy individuals and UC microbiota, as well as the inflamed or non-inflamed intestinal tract in UC (Sokol et al., 2017). *Ascomycota* and *Basidiomycota* may be involved in the intestinal tract inflammatory process or driven by disease. Interestingly, we also found significant changes in taxa at genera (such as *Cryptococcus*, *Nakaseomyces*, and *Encephalitozoon*) and species (such as *Fusarium oxysporum*, *Candida glabrata*, and *Encephalitozoon hellem*) levels with low abundances. These low-abundance taxa might be associated with the disease and deserve further exploration. However, since the total fungal reads per sample obtained in this study were relatively limited, future studies with deeper fungal sequencing depth and bigger sample sizes are needed to confirm this result of significant taxa with low abundances, as well as to study the mycobiome.

Different fungal fecal flora was found in donor individuals and UC patients before and after capsule FMT. Most fungal species have been isolated from the intestinal contents, including *Candida*, *Saccharomyces*, *Trichosporon Cryptococcus*, *Galactomyces*, *Penicillium*, *Yarrowia*, and *Debaryomyces (* Haupt et al., 1983; Galan-Sanchez et al., 1999; Khatib et al., 2001; Silva et al., 2004; Jain et al., 2021). Among these, *Cryptococcus*, *Saccharomyces, Debaryomyces*, and *Candida* were significantly altered in patients who achieved Rm after capsulized FMT. Other species were originally cultured from other habitats, including the skin and respiratory tract (Maesaki et al., 1993; Timm et al., 2020). Some fungal species, such as *Candida*, *Fusarium*, and *Debaryomyces*, have been reported as opportunistic pathogenic fungal colonic microbiota in the disease and are related to disease status.

*Candida* can thrive in the gut, respiratory and urogenital tract, vagina, oral mucosa, and skin of humans (Vautier et al., 2015). A high abundance of *Candida* species is observed in Crohn’s disease (CD) and UC (Li et al., 2014; Hoarau et al., 2016; Liguori et al., 2016; Sokol et al., 2017). Moreover, different *Candida* species have been involved in UC pathogenesis, including *C. albicans* (Chehoud et al., 2015; Mukherjee et al., 2015; Richard et al., 2015) and *C. glabrate (* Gouba and Drancourt, 2015; Sierra-Diaz et al., 2019). In this study, we did not find significant changes in *C. albicans* which might be due to the heterogeneity of *C. albicans* in patients using our shotgun sequencing approach. However, we found a decreased abundance of *C. dubliniensis* and *C. glabrate* was associated with clinical Rm after capsulized FMT*. C. dubliniensis* is phenotypically similar to *C. albicans* and is increasingly recognized as an opportunistic pathogenic fungus in immunocompromised hosts (Gutierrez et al., 2002; Jewtuchowicz et al., 2008; Yamahiro et al., 2016; Tahir et al., 2020). *C. albicans* can break through the mucosal barrier and induce an intestinal immune response related to UC pathogenesis (Naglik et al., 2011; Romani, 2011). *C. dubliniensis* also has the potential to break through the mucosal barrier. A genome-wide inventory of peptide transport (PTR) transporters identified two PTR transporters in *C. dubliniensis (* Khatoon et al., 2022). Madiha (Tahir et al., 2020) also cultured *C. dubliniensis* in the cerebrospinal fluid from a 27-year-old patient with chronic meningitis and chronic hepatitis C. Unlike *C. albicans* or *C. dubliniensis*, *C. glabrate* grows only as blastoconidia *(yeast)* and has been reported to be overrepresented in CD (Liguori et al., 2016). Notably, a major group of adhesions in *C. glabrate* is encoded by the epithelial adhesin gene family, which enables *C. glabrate* to adhere to epithelial cells, causing mucosal infections (Cormack et al., 1999; De Las Penas et al., 2003).

In addition, we observed a significant decrease in *Fusarium* spp. in patients who achieved Rm after capsulized FMT. *Fusarium* spp. can produce beauvericin and moniliformin. These secondary metabolites are mycotoxins found in cereal samples (Kamyar et al., 2006). *In vitro*, beauvericin can increase the intracellular calcium concentrations leading to eryptosis. Fumonisins is also one of mycotoxins produced by *Fusarium* spp. that are considered potentially carcinogenic mycotoxins in humans (Woloshuk and Shim, 2013; Munkvold, 2017). Notably, the relative abundance of *D. hansenii* was persistently decreased in patients who achieved clinical Rm after capsulized FMT from W4 to W12. In a previous study (Jain et al., 2021), *D. hansenii* was localized in intestinal inflammatory wounds and impaired colonic healing in CD and colitis mice.

In conclusion, this study identified fungal dysbiosis in patients with UC and the specific fungal taxa associated with therapeutic outcomes in patients with UC receiving capsulized FMT. Our findings, such as fungal community diversity and removal of *Candida*, *Fusarium*, and *Debaryomyces* species, may provide new insights into FMT in UC and provide reference information about therapeutic microbial manipulation of FMT to enhance its effects.

## Data availability statement

The datasets presented in this study can be found in online repositories. The names of the repository/repositories and accession number(s) can be found below: https://www.ncbi.nlm.nih.gov/, PRJNA672846.

## Ethics statement

The studies involving human participants were reviewed and approved by ClinicalTrails.gov (NCT03426683). The patients/participants provided their written informed consent to participate in this study.

## Author contributions

QC, YF, BZ, JR and HX designed this study. QC, YF, BZ underwent the FMT treatment and analyzed the data. LW, YH, QH, JS were responsible for patients’ management. ZC and CY were responsible for donors’ management. QC and BZ wrote the manuscript.
