# Supplementary figures and images for: Specific fungi associated with response to capsulized fecal microbiota transplantation in patients with active ulcerative colitis
Source: Front Cell Infect Microbiol. 2023 Jan 5;12:1086885. doi: 10.3389/fcimb.2022.1086885 (PMC9849685; doi:10.3389/fcimb.2022.1086885)

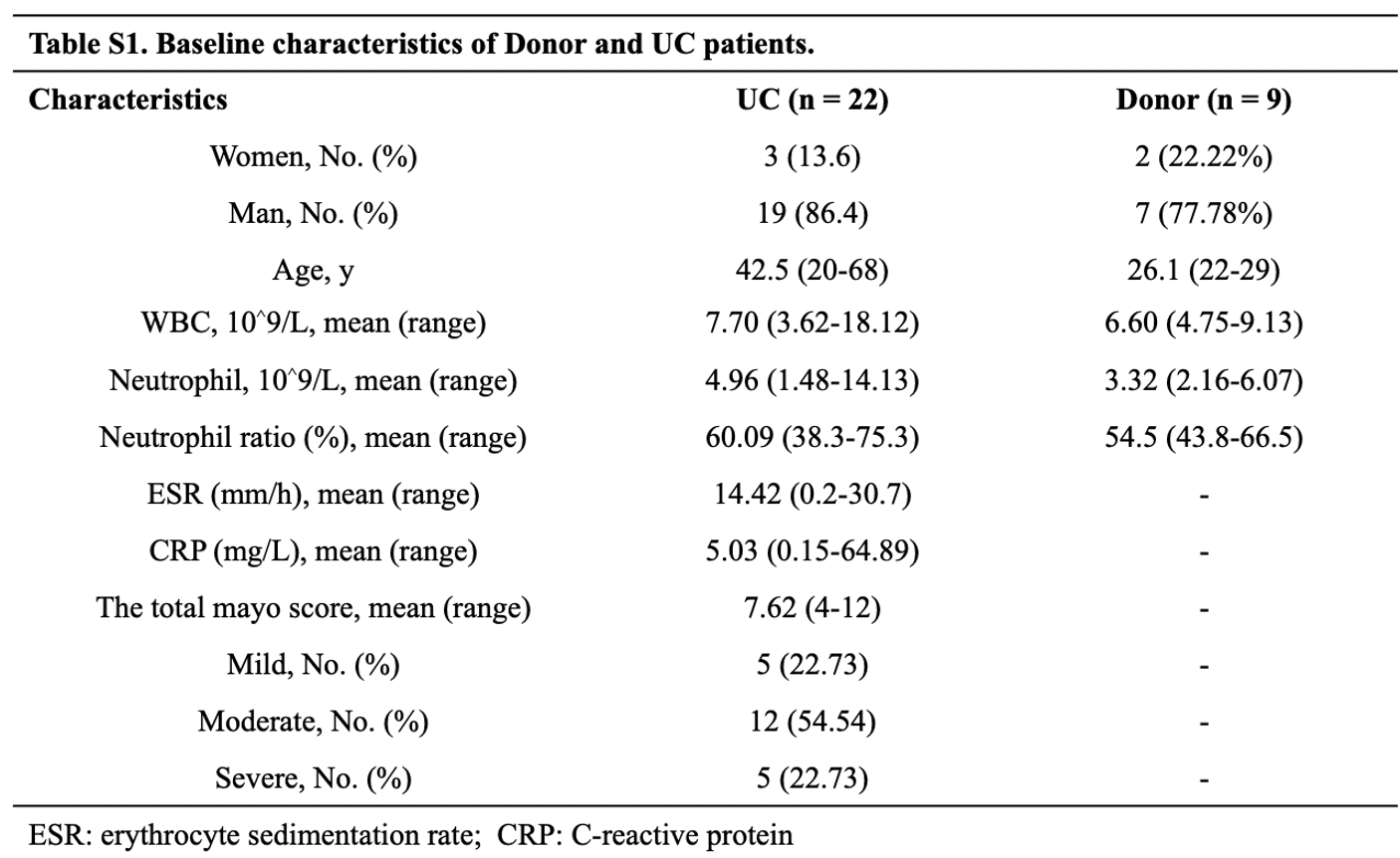

Supplement: Supplementary file 1 [file Image_1.jpeg]
