# Supplementary material for: Specific fungi associated with response to capsulized fecal microbiota transplantation in patients with active ulcerative colitis
Source: Front Cell Infect Microbiol. 2023 Jan 5;12:1086885. doi: 10.3389/fcimb.2022.1086885 (PMC9849685; doi:10.3389/fcimb.2022.1086885)
Supplement: Supplementary file 2 [file DataSheet_1.docx]

**Supplementary materials**


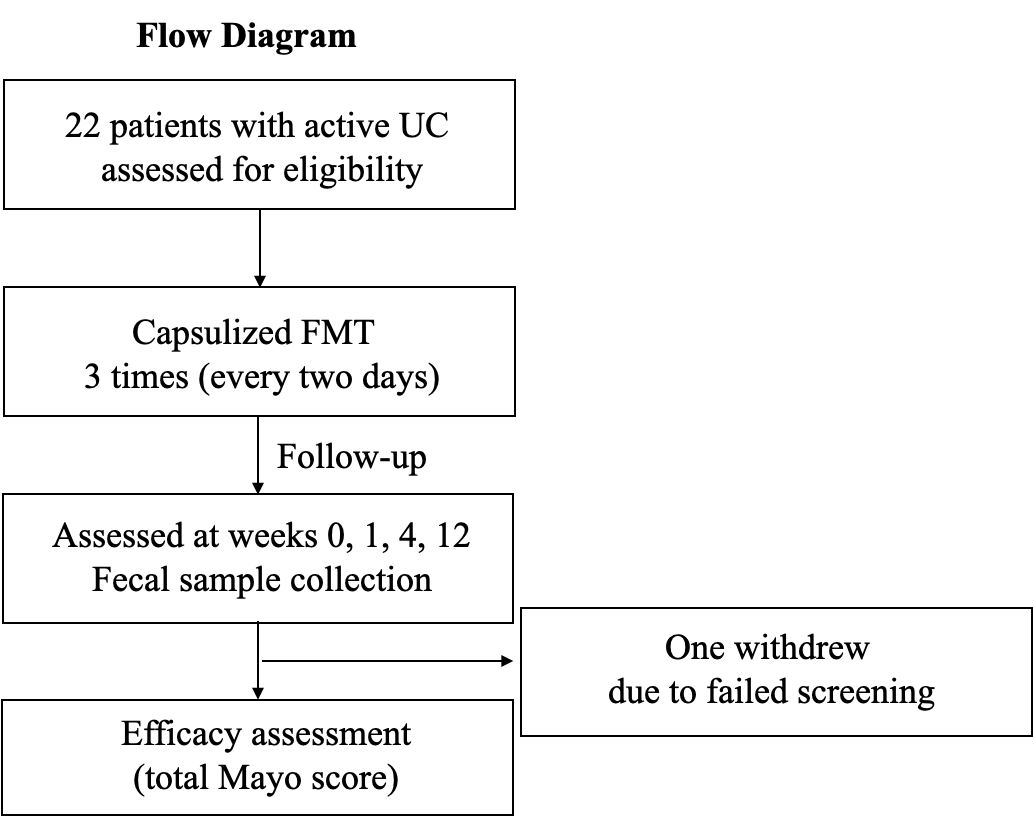


**Figure S1. Flow diagram.**


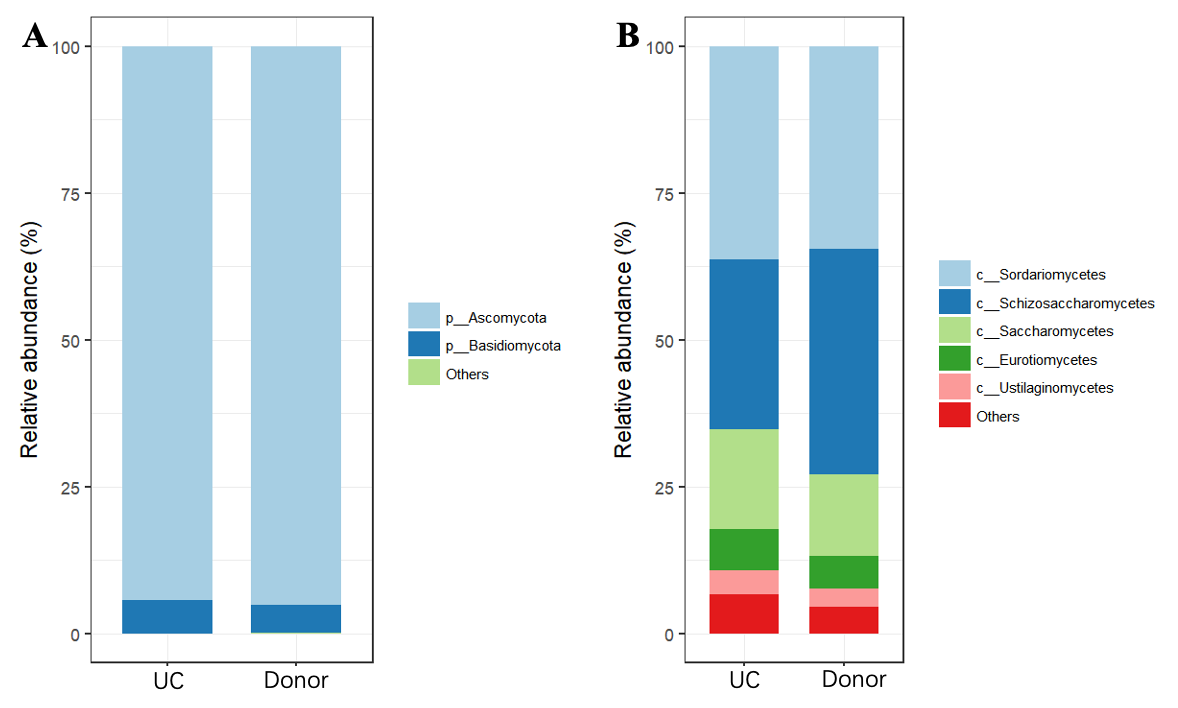


**Figure S2.** Changes in overall fungal community structures at the phylum level (A) and class level (B) in fecal samples collected from donors and patients.


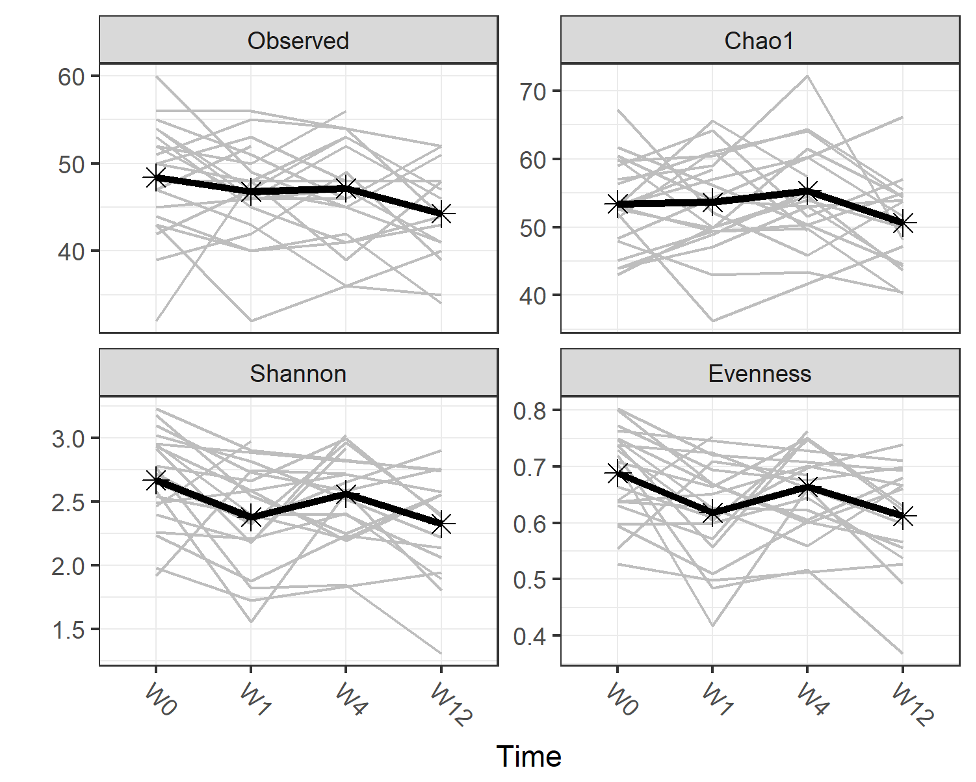


**Figure S3.** **Differences in gut fungal communities after capsulized FMT treatment in different times on OUT level.** Changes of alpha-diversity indexes estimated by richness (Observed OTUs, Chao1), Shannon, and Pielou’s evenness of each individual over time. The star indicate the mean value of each timepoint.


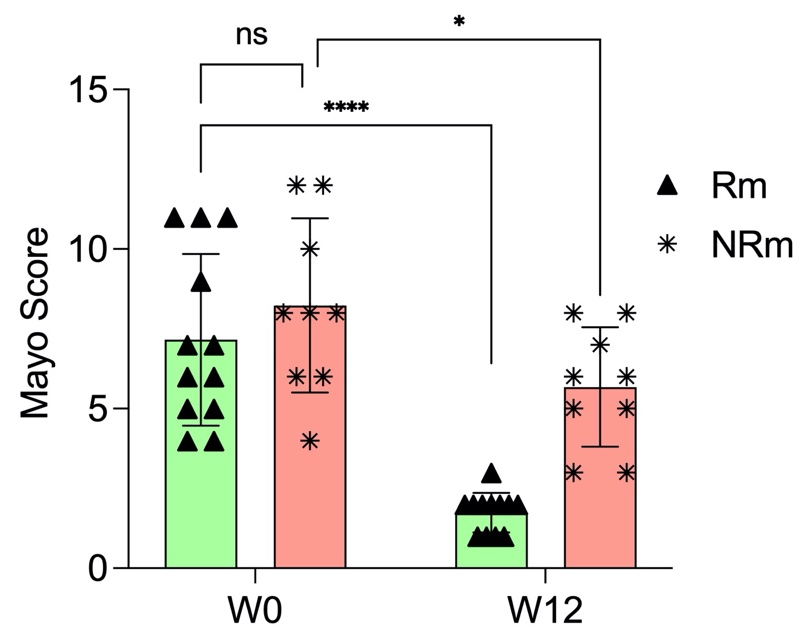


**Figure S4. Alteration in the total Mayo score of the Rm/NRm group before and after capsulized FMT.** Paired t-test was used between W0 and W12 in Rm/NRm group. Wilcoxon test was used in Rm and NRm groups at W0. The asterisk indicated significant differences between the two groups. **p* $\leq$0.05,***p* $\leq$0.01, ****p* $\leq$0.001.
